# Engaging Stakeholders to Implement an Aging in Place Intervention through an Area Agency on Aging

**DOI:** 10.1093/geroni/igaa057.3228

**Published:** 2020-12-16

**Authors:** Pamela Toto, Caylee Yanes, Molly Ennis, Beth Fields

**Affiliations:** 1 University of Pittsburgh, Pittsburgh, Pennsylvania, United States; 2 University of Wisconsin–Madison, Madison, Wisconsin, United States

## Abstract

Community Aging in Place, Advancing Better Living for Elders (CAPABLE) is an interdisciplinary evidence-based intervention for reducing disability, reducing cost, and promoting aging in place for older adults who qualify for Medicaid services. However, implementation of CAPABLE through existing community services and with different older adult populations may require adaptations based on stakeholder input. The purpose of this qualitative study was to adapt CAPABLE for implementation through an Area Agency on Aging (AAA) targeting older adults with disability but who do not meet thresholds for Medicaid services. Data collection occurred with stakeholders from a single AAA. Two, 60-minute focus groups were conducted with frontline providers (n=7) and administrators (n=7). Thematic analysis of the data were completed using NVivo 12 Pro. Stakeholders described three themes to consider for implementing CAPABLE in their AAA: screening and referral process, eligibility, and team meetings. Frontline providers recognized the need to allow care managers “to decide who would be most appropriate for the program because they have a better understanding of the person, their family, and home environment.” Administrators supported expanding eligibility requirements, “Could we offer the program to someone even if they were recently hospitalized?” All stakeholders expressed that an initial interdisciplinary team meeting could be beneficial for goal setting, “If the team and consumer could be together for the first meeting that could save time and help generate ideas for goals.” These perspectives affirm the importance of early stakeholder engagement in adapting to adopt evidence-based programs into “real-world” settings.

